# Influence of hole shape/size on the growth of site-selective quantum dots

**DOI:** 10.1186/1556-276X-8-504

**Published:** 2013-12-01

**Authors:** Christian J Mayer, Mathieu F Helfrich, Daniel M Schaadt

**Affiliations:** 1 Institute for Energy Research and Physical Technologies, Clausthal Technical University, Am Stollen 19B, Goslar 38640, Germany; 2 DFG-Center for Functional Nanostructures (CFN), Karlsruhe Institute of Technology (KIT), Wolfgang-Gaede-Straße 1a, Karlsruhe 76131, Germany

**Keywords:** Molecular beam epitaxy, MBE, QD, Quantum dot, InAs, Site selective, Lithography, Dry etching, ICP

## Abstract

The number of quantum dots which nucleate at a certain place has to be controllable for device integration. It was shown that the number of quantum dots per nucleation site depends on the size of the hole in the substrate, but other dimensions of the nucleation site are vague. We report on the influence of hole shape on site-selectively grown InAs quantum dots (QDs) by molecular beam epitaxy. Dry etching of the GaAs wafers was used because of its high anisotropic etching characteristic. Therefore, it was possible to verify the influence of several hole shape parameters on the subsequent QD growth independently. We show that the nucleation of these QDs depends on several properties of the hole, namely its surface area, aspect ratio of the surface area, and depth. Especially, the aspect ratio shows a big influence on the number of nucleating QDs per site. With knowledge of these dependencies, it is possible to influence the number of QDs per site and also its distribution.

## Background

Quantum dots (QDs) can be formed by growing InAs on GaAs by molecular beam epitaxy (MBE) in the Stranski-Krastanov growth mode [1-6]. The finite lattice mismatch between the two materials leads to the formation of nanometer-scaled InAs islands which, if covered with GaAs, act as QDs due to the lower bandgap of InAs [7,8]. These QDs show unique properties which make them interesting for many applications like single photon sources [9-13].

For device fabrication, it is sometimes required to place QDs at certain locations. For example, in a microcavity, the QDs have to be placed exactly at the mode positions of photonic cavities in order to maximize coupling and therefore device performance [13]. The positioning of QDs can be achieved by modification of the GaAs surface in the nanoscale. Electron beam lithography (EBL) [4-6,14], local oxidation [15-17], focused ion beam [18], or nanomechanical stamping [19] can be used to fabricate small holes on the substrate surface. The deformation of surface chemical potential leads to accumulation of In adatoms in the holes which then act as preferential nucleation sites for InAs QDs. These pre-patterning techniques come with disadvantages due to surface degregation in terms of defects and impurities, which can limit the performance of the optical quality of the quantum dots. Nevertheless, it was shown that with an appropriate treatment, such as efficient sample cleaning [20], multistacking [21], or partial capping [22], good optical qualities can be achieved, e.g., small linewidths down to 100 *μ*eV for single-layer QDs [20] or even 43 *μ*eV for certain single QDs [22].

QD nucleation can be controlled by several methods. In prior works, we investigated the influence of hole spacing and post-growth annealing [23,24]. It was also shown by other groups that growth parameters like flux [25], InAs deposition [26], and growth temperature [27,28] can influence the nucleation. In this work, we focused on the effects of hole geometry and fabrication, such as hole size, shape, and depth, on the subsequent growth of site-selective QDs. Improving and adapting these parameters provide an additional control mechanism and might lead to further optimization. We used EBL combined with dry etching in our work as this is the most versatile patterning technique and therefore allows changing various pattern parameters easily. Dry etching showed superior controllability compared to the previously used wet chemical etching (WCE) [24,29] as it is able to influence the hole shape and size much better due to a highly anisotropic etching [30,31]. While hole size is known to influence the number of nucleating QDs [5] and post-growth techniques such as *in situ* annealing have been shown to modify these [24], knowledge on the influence of other hole parameters like aspect ratio or depth remained vague.

## Methods

**Figure 1:**
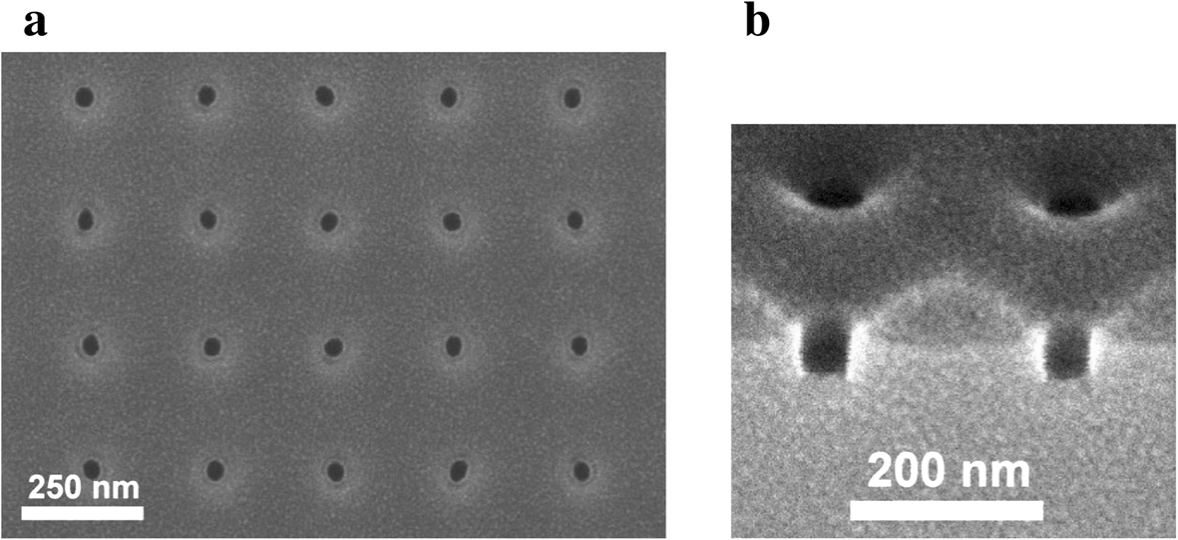
**Top and profile images of dry etched-holes.** SEM images of holes after dry etching with resist remaining on the surface. Regularly shaped circular holes are observed in the top view **(a)** while the profile in **(b)** shows the vertical sidewalls. The resist is affected near the holes and pushed back. Therefore, the holes increase with etching time in lateral dimension.

The samples were grown in a Riber Compact 21T MBE system (Riber, Paris, France) on (1 0 0) epi-ready GaAs. A 300 nm thick buffer layer was grown at a temperature of 580°C in order to flatten the surface and to get a reproducible starting point before coating the samples with an 80 nm thick layer of polymethyl methacrylate with methacrylic acid (PMMA/MA). The resist was exposed in a Supra 55VP from Zeiss (Oberkochen, Germany) with lithography attachment provided from Raith (Dortmund, Germany) at an accelerating voltage of 30 kV. Afterwards, the samples were developed using a solution composed of 2:3 methyl isobutyl ketone (MIBK)/isopropanol, hard baked at 130°C for 30 min and then dry etched by reactive ion etching (RIE) using an inductively coupled plasma (ICP) in an ICP 180 from Oxford Instruments (Abingdon, UK). Before each etching run, the chamber was cleaned with oxygen plasma for at least 30 min until the plasma and the direct current (DC) bias were stable. After inserting the sample and a small temperature stabilization step at 10°C, the plasma was ignited at a pressure of 5 mTorr. The sample was then etched with 2:4 sccm SiCl_4_/Ar at the lowest reachable pressure of 1.9 mTorr in order to decrease the etching rate [32,33]. Vertical sidewalls could be produced using a 20 W radio frequency forward power (≈50 V DC bias) and a 150 W ICP power, as demonstrated in Figure 1.

Using this etching recipe, the depth and shape of the holes can be influenced separately, and also, the shape of the hole in the resist is transferred almost 1:1 into the underlying GaAs substrate. After etching, the resist was removed by an adequate remover mainly consisted of acetone, followed by cleaning with different solvents (trichlorethylene, acetone, *n*-methyl-2-pyrrolidone) and dipping in a heated ultrasonic bath (isopropyl acolhol, methanol, ethanol), as also performed in prior studies [29]. The cleaning procedure was finalized with a 35 min plasma asher treatment in oxygen atmosphere and a 10 s dip into diluted hydrochloric acid. A 12 nm thin GaAs buffer layer is deposited followed by a small annealing step for 20 s in order to reduce surface roughness created during etching. The beam equivalent pressures were ≈8×10^-9^ bar for As and ≈3.5×10^-10^ bar for Ga. The InAs QDs are grown for 24 s, which is equivalent to 1.5 ML. For all steps, the substrate temperature was held at 500°C. The influence of the hole properties, e.g., the hole shape, was then investigated by comparing the amount of QDs nucleated in the holes. Information on these properties were obtained from scanning electron microscopy (SEM) images using the image analysis tool ImageJ (NIH, Bethesda, MD, USA) [34]. The depth of the holes was obtained from atomic force microscopy (AFM) scans.

## Results and discussion

**Figure 2:**
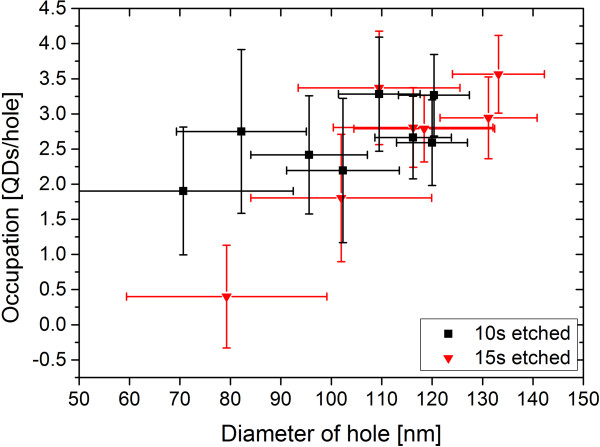
**Dependence of the nucleating QDs per hole on the diameter.** The number of QDs that nucleate inside a hole is dependent on the hole diameter. The larger the hole, the more QDs nucleate within such a hole, which can be explained by the larger nucleation site and therefore the larger available In reservoir.

At first, the influence of the hole size on the nucleation of QDs per hole (occupation) was investigated and is shown in Figure 2. The hole diameters were calculated from the surface area of the holes which was extracted from SEM images by ImageJ. The original hole sizes were equal for all three etching times (10, 15, and 20 s), but lateral etching leads to larger holes at longer etching times due to the push back of the resist as demonstrated in Figure 1. Despite strong size fluctuations, which possibly resulted from imperfections of the electron beam exposure, an increase of QD occupation is observed for larger hole diameters. This is in agreement with the work of Jeppesen et al. [5].

**Figure 3:**
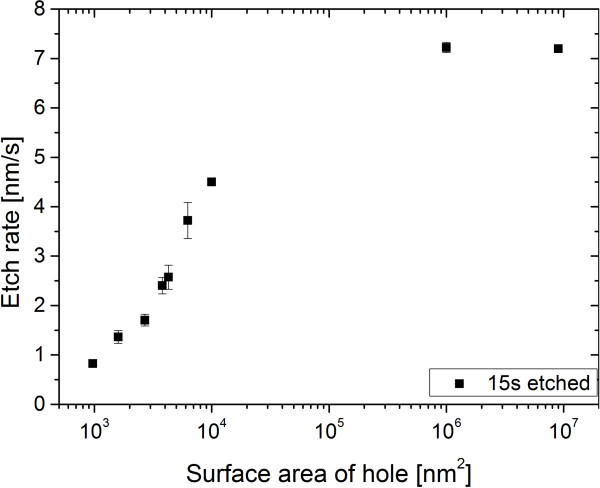
**Etching rate dependence on the surface area of the holes.** The etching rate is dependent on the surface area of the holes and it is increasing strongly for small structures. For very large structures, the etching rate converges to an independent value, which is eight times higher than for the smallest investigated structures.

Assuming the same attractive force to accumulate In adatoms for holes of all size, the larger ones will contain more InAs and therefore allow more QDs to form. Due to the dense pattern together with the given amount of deposited InAs, it is expected that the holes are not maximally filled with QDs so that the difference in occupation is only related to the accumulated amount of material and not limited by diffusion [23]. A higher standard deviation of the average QD occupation is found for smaller holes. This is possibly related to the fact that the absolute accuracy with which holes are defined in the resist during EBL yields a larger relative size fluctuation for smaller holes. Since the etching rate for a nanohole depends on its opening, i.e., its lateral size, see Figure 3, small size fluctuations in the resist get amplified during dry etching. Measurement errors by the program ImageJ that has to distinguish between the plane surface and the hole surface gain importance for smaller holes. Since the size of the holes is relatively large, this contribution should not be very high though.

In addition, it can be seen that the occupation increases more strongly for the 15 s etched sample. While the average number of QDs per hole seems to be lower for the 15 s sample compared to the 10 s sample for small holes, for holes larger than 120 nm, the occupation seems to be equal or even higher for the longer-etched sample. The reason for such behavior must be related to the increased depth of the holes because the increase in lateral size due to chemical etching does not lead to an expected higher occupation. Therefore, besides the lateral size, the shape of the hole influences the number of nucleating QDs.

**Figure 4:**
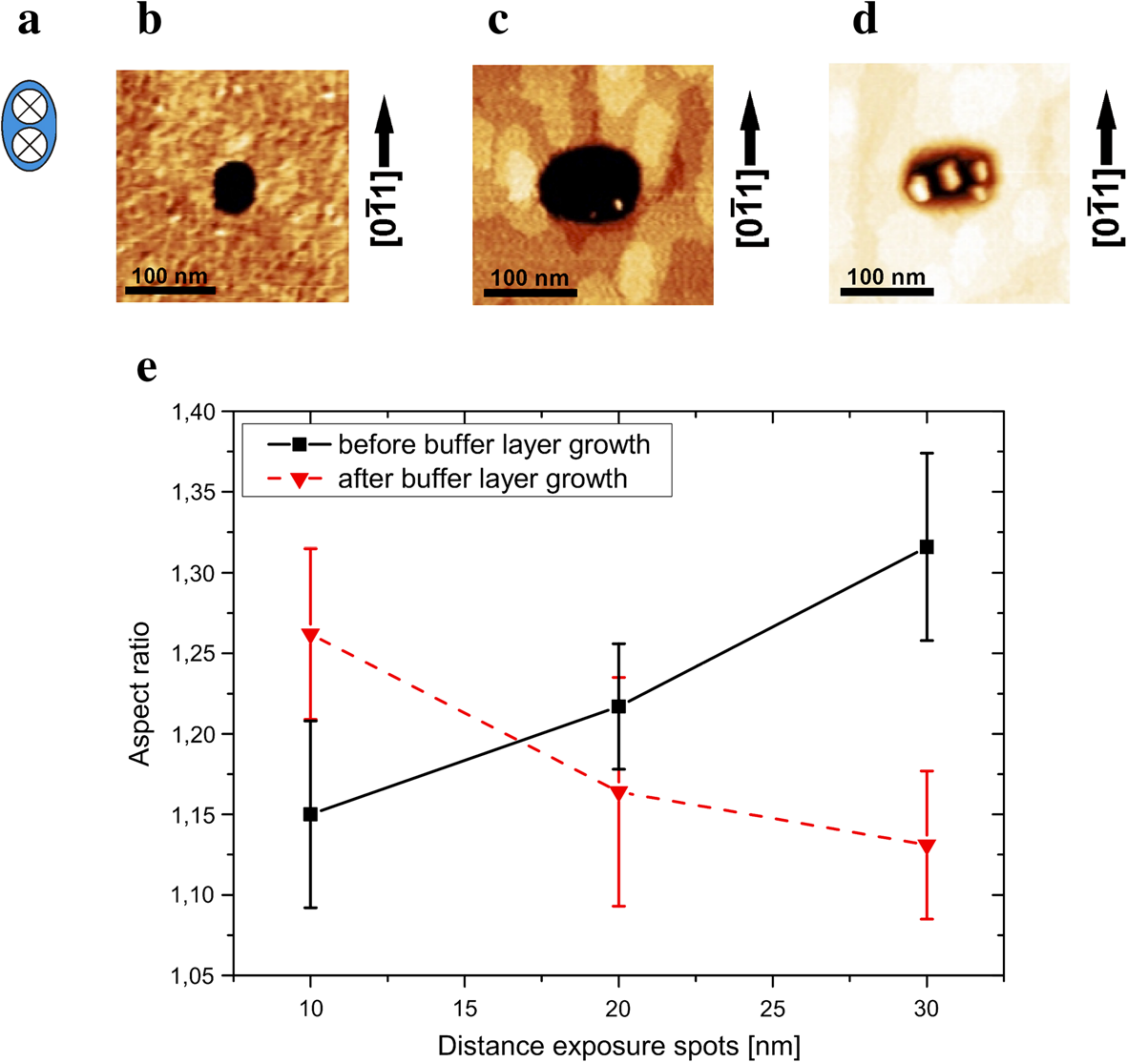
**Manipulation of the aspect ratio by appropiate exposure design.** Comparison of the aspect ratio before and after the buffer layer growth. Two dots with a certain distance are exposed to the resist **(a)** in order to define an elongated structure, see **(b)**. The attachment of GaAs depends strongly on the crystallographic direction leading to an elongated structure perpendicular to the previous one, see **(c)**. This elongation leads to a nucleation of QDs along a chain, see **(d)**, and is therefore undesired. With increasing distance of the two exposure spots, it is shown in **(e)** to increase the aspect ratio before the buffer layer growth and therefore decrease the aspect ratio after the buffer layer growth due to the different migration rates.

The shape of the written structure in the resist is preserved during dry etching and hence can be investigated. The overgrowth of holes depends on crystallographic direction so that elongated/elliptical shapes are obtained after overgrowing originally circular holes with a thin GaAs buffer layer. Different migration rates in the 〈0 1 1〉 and $\langle 0\;\bar{1}\;1\rangle$ axes are responsible for this shape transformation, see Figure 4[35-38]. Since it is not possible to balance these different migration rates, a different approach was developed. In order to get a circular hole and thus an isotropic nucleation site, an elongated structure is written into the resist with the elongation being perpendicular to the one observed after buffer layer growth. The easiest way to create elongated structures is by exposing two single spots close to each other, see Figure 4a. If close enough, the two exposure spots will merge into an ellipse. In our work, the distance between the exposure spots was varied from 10 to 30 nm. The elongated structures were arranged on a square grid with 500 nm spacing. The elliptical holes are elongated along $\left[0\;\bar{1}\;1\right]$ after etching (Figure 4b). After overgrowing the holes with a GaAs buffer layer, the effective migration of Ga adatoms to As-terminated facets leads to an elongation of the defined structure in the [0 1 1] direction (Figure 4c). Thus, the initial elongation is compensated by the buffer layer growth and the final hole becomes more symmetric. Hence, the aspect ratio (major axis /minor axis) after buffer layer growth decreases with increasing separation of the two exposure spots. Using this approach, it was possible to reduce the aspect ratio of the final hole from, e.g., 1.26±0.05 to 1.13±0.05 for the 20 s sample. Reducing the aspect ratio is promising due to the alignment of the QDs inside the hole as they align along a chain (Figure 4d) in the direction of the hole elongation, i.e., [0 1 1] [37,39].

The result of writing ellipses instead of round holes into the resist is shown in Figure 4e. The aspect ratio of the major elliptical axes is given with respect to the separation of the two exposure spots before buffer layer growth (black) and after buffer layer growth (red). As intended and shown in Figure 4, the aspect ratio increases (decreases) with increasing distance of the two exposure spots before the buffer layer growth (after the buffer layer growth).

Next, the influence of the aspect ratio on the QD nucleation was investigated. Two samples, dry etched for 10 and 15 s, are compared. With increasing distance between the two exposure spots, the final aspect ratio decreases, while the hole size increases. This effect can be seen for both samples. The differences in hole size between the two samples emerge as mentioned above. Longer-etched holes become larger due to a pullback of the resist near the holes by sputtering from the etching gases (compare Figure 1 where the resist is affected near the holes).

Furthermore, the aspect ratios of longer-etched holes are smaller. This might be explained by insufficient optimization of the etching gas parameters. The used etching gas is composed of two gases, SiCl_4_ and Ar. With these two selected etching gases, there is a chemical component (from the SiCl_4_) and a sputter component (mainly Ar). The resulting etching characteristic then depends on the gas mixture and selected powers. Chemical etching of GaAs in the $\left[0\;\bar{1}\;1\right]$ direction is usually two to five times faster than in the perpendicular [0 1 1] direction, therefore increasing the effect of the separated holes.

**Figure 5:**
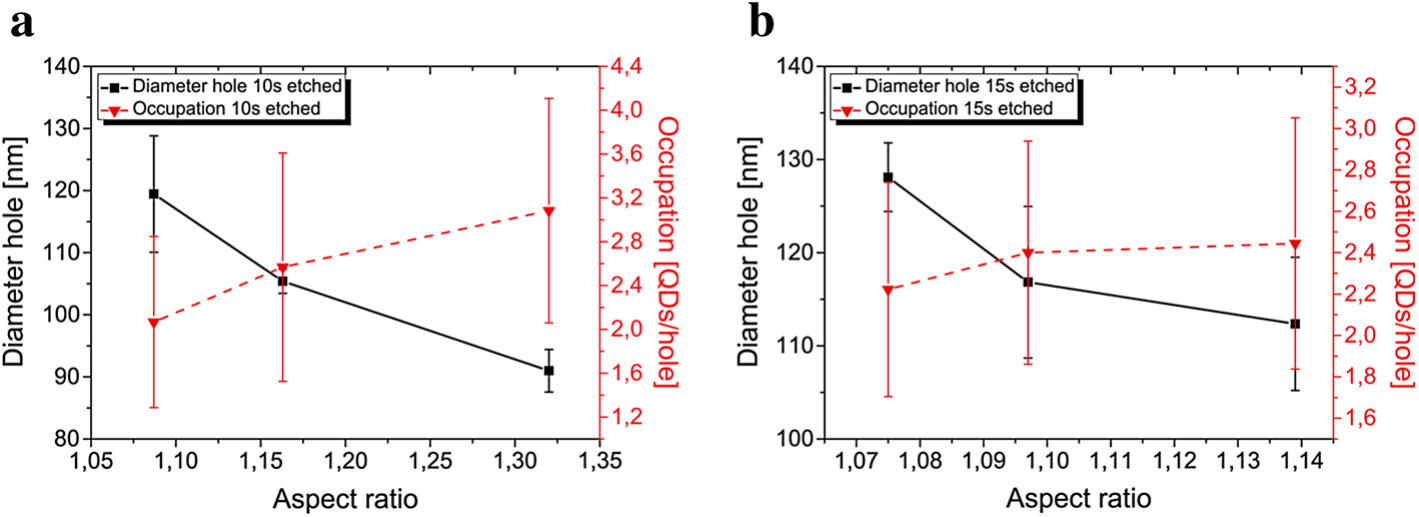
**Influence of the aspect ratio on the hole occupation.** The influence of the occupation and diameter of the holes depending on the aspect ratio is given for 10 **(a)** and 15 s **(b)** of etching time. With this basic approach of two separated exposure spots, the diameter of the holes increases with decreasing aspect ratio. The advantage of a hole with smaller aspect ratio therefore comes with a disadvantage of a larger hole. Nevertheless, a smaller number of QDs per hole nucleate with decreasing aspect ratio but larger hole size. This can be seen for both etching times shown. Increasing the etching time leads to larger holes as seen before, but smaller aspect ratio and thus smaller occupation.

The hole occupation is given with respect to the aspect ratio in Figure 5. For both etching times, the number of QDs per hole increases with increasing aspect ratio. Compared to the results in Figure 2, this is a bit surprising because the number of QDs per hole decreases with decreasing aspect ratio although the hole diameter is strongly increasing. Apparently, the tendency of higher occupation numbers for larger holes is influenced by the aspect ratio of the holes. Therefore, it is possible to decrease the occupation by using larger holes with smaller aspect ratios.

At last, the influence of the etching depth is investigated. The etch rate depends strongly on the size of the etched structure, see Figure 3. At first, it increases very strongly with the hole area, which is due to the supply shortage of the etching gases through the small hole size. With increasing size of the etched structure, this effect becomes negligible and the etch rate converges to the etch rate of a free surface. The largest structures show about an eight times higher etching rate than the smallest investigated structures, which has to be taken into account if structures with different sizes are etched at the same time.

**Figure 6:**
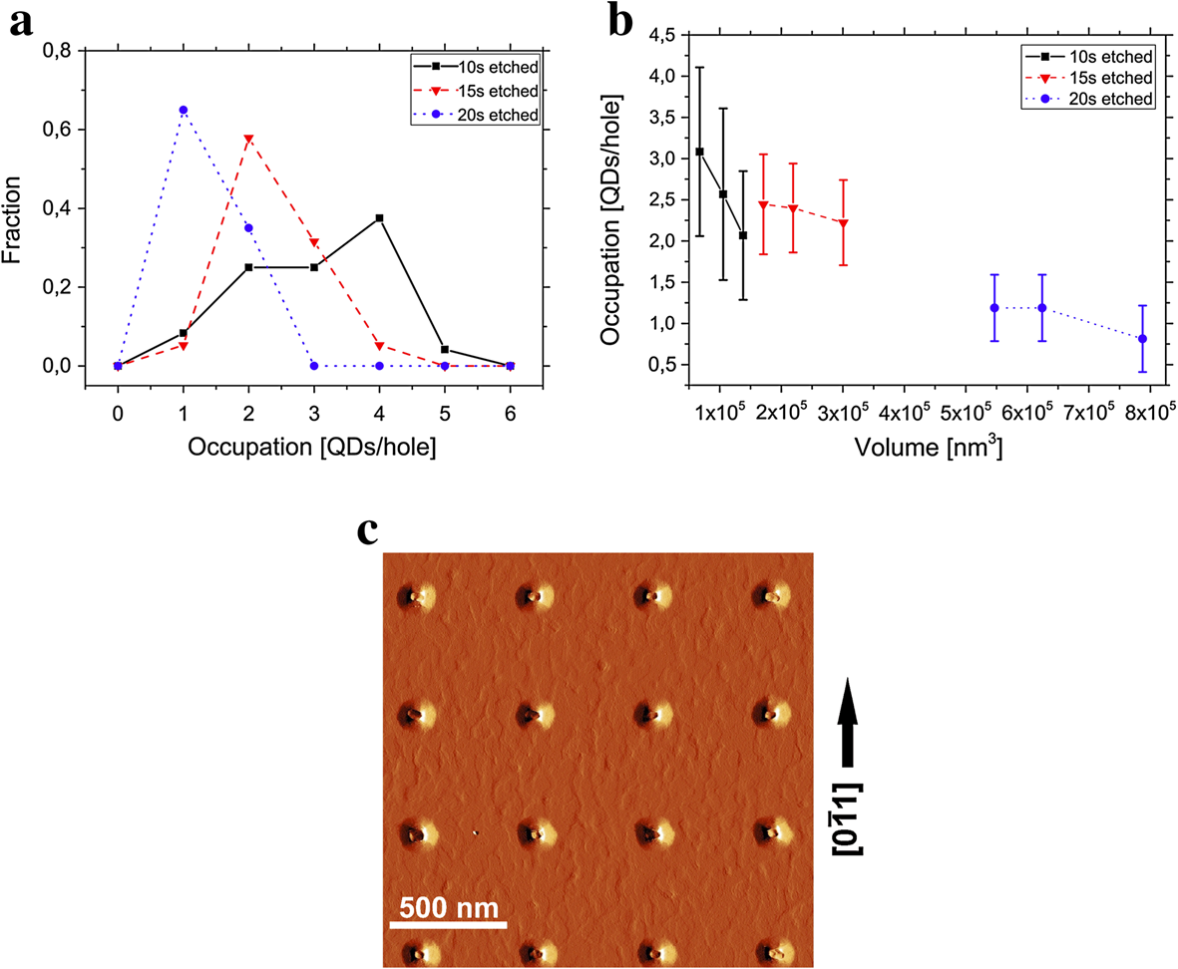
**Influence of depth on the amount of nucleating QDs per holes.** In **(a)**, the fraction of the number of QDs per hole nucleating inside a hole is given. With increasing etching duration and therefore depth, the number of QDs per hole decreases. Also, the broadening of the distribution and therefore the standard deviation of the number of QDs per hole is smaller. The influence of the volume of the hole on the number of QDs nucleating per hole is given **(b)**. Both images show the superior properties of deeper holes. In **(c)**, an amplitude picture of an AFM scan is given. It can be seen that although the diameter is quite large with a size of 150.3±4.1 nm and an aspect ratio of 1.164±0.071 is also not perfect, the number of QDs can be decreased to one to two QDs per hole. Optimizing these parameters should therefore lead to a number of QDs closer to one.

The influence of depth on the occupation is investigated next. The 20 s etched holes were too deep for SEM investigation, and therefore, AFM images were used for all samples in Figure 6. The distribution of occupation numbers is shown for three different etching times for an initially equal hole size inside the resist.

The 20 s etched sample has a maximum at one QD per hole of about 0.6. This means that 60% of all holes are occupied with one quantum dot. With decreasing etching depth, the maximum of the distribution is heading to a higher number of QDs per hole. Also, the distributions get broader for smaller etching depths, meaning that the average number of QDs per hole has a larger standard deviation. This behavior was seen for all investigated hole sizes and also hole spacings. This is remarkable because the size of the holes increases with increasing etching time, as seen before, which should increase the number of QDs for the longer-etched samples. The influence of depth can also be seen in Figure 6b where the number of QDs is given with respect to the volume of the holes. Since the depth and lateral size cannot be fully adjusted separately, the volume of the holes is given. It is calculated by the lateral size and depth of the holes. Despite the fact that the holes gain size, the influence of depth is dominant, and with increasing depth, fewer QDs nucleate within one nucleation site. At last, one AFM image of a 20 s etched sample is shown in Figure 6c. Two separated exposure spots with a distance of 20 nm were used in order to decrease the aspect ratio. The picture shown is an amplitude picture of this sample in order to also show the nucleated QDs inside the holes. As can be seen, there is still a small elongation of the holes with an aspect ratio of 1.16 ± 0.07 in the [0 1 1] direction and the holes are large with a diameter of 150.3±4.1 nm. Although the aspect ratio and diameter of the holes might be optimized further, the sample shows only a small number of QDs of one to two per hole. Decreasing of aspect ratio and diameter and increasing of hole depth might therefore lead to even smaller values of occupation.

## Conclusions

The number of quantum dots which nucleate at a certain place has to be controllable for device integration. We investigated the influence of the size, aspect ratio, and depth of the nucleation site on quantum dot nucleation. The occupation increases with increasing aspect ratio, where the QDs align along a chain in the elongated direction. Increasing the distance of two separated exposure spots in the $\left[0\;\bar{1}\;1\right]$ direction leads to a decrease of holes after the buffer layer growth. We showed that a smaller aspect ratio has an advantageous effect on the QD growth, which is not compensated by the worsening influence of the increased nucleation site. The depth of the nucleation site decreases the average number of QDs nucleating inside a hole and also seems to decrease its standard deviation. We showed that the nucleation of QDs can be influenced by the size, shape, and depth of the nucleation site. With *in situ* annealing, this should provide another possibility of influencing and optimizing the number of QDs within a nucleation site. The strong dependence of the etching rate on the structure size was also shown.

## Abbreviations

AFM: Atomic force microscopy; EBL: Electron beam lithography; ICP: Inductively coupled plasma; MBE: Molecular beam epitaxy; MIBK: Methyl isobutyl ketone; PMMA/MA: Polymethyl methacrylate with metacrylic acid; QD: Quantum dot; RIE: Reactive ion etching; SEM: Scanning electron microscope.

## Competing interests

The authors declare that they have no competing interests.

## Authors’ contributions

CJM prepared the samples by EBL and ICP-RIE, carried out the AFM and SEM measurements, analyzed the data, and drafted the manuscript. MFH carried out the MBE growth of the samples, gave support in data evaluation and interpretation, and helped draft the manuscript. DMS conceived of the study and participated in its design and coordination. All authors read and approved the final manuscript.
